# Supplementary material for: Longitudinal proteomic profiling of cerebrospinal fluid in untreated multiple sclerosis defines evolving disease biology
Source: Nat Commun. 2025 Dec 3;16:11012. doi: 10.1038/s41467-025-65154-8 (PMC12695886; doi:10.1038/s41467-025-65154-8)
Supplement: Supplementary file 1 — Supplementary Information [file 41467_2025_65154_MOESM1_ESM.pdf]

Longitudinal proteomic profiling of cerebrospinal fluid in untreated multiple sclerosis defines evolving disease biology

Peter Kosa, Shinji Ashida, Keith Lumbard, Jing Wang, C. Jason Liang, Ruturaj Masvekar, Yujin Kim, Mihael Varosanec, Lori Jennings, Bibiana Bielekova

Supplementary notes

Note 1: Definitions of selected terminology

Tissue remodeling - denotes structural changes in CNS tissue architecture mediated by stromal cells in response to injury. These changes are aimed at limiting further damage to resident CNS cells such as neurons and glia. Remodeling may involve repair of the blood- brain barrier or brain-epithelial barrier through increased secretion and altered composition of extracellular matrix (ECM) components—such as fibrillar collagens, fibronectin, laminin, versican, and hyaluronan—leading to perivascular fibrosis. It may also involve proliferation and de-differentiation of stromal cells into pro-fibrotic myofibroblasts, and phenotypic shifts toward inflammatory fibroblasts that produce chemokines, upregulate adhesion molecules, and promote immune cell recruitment.

Non-lesional damage - refers to alterations in central nervous system (CNS) tissue architecture that are not associated with the formation of new MS lesions or with the quantifiable change in T2LL.

Stromal cell interaction - refers to the physiological roles stromal cells play in maintaining CNS tissue integrity and supporting neuronal function. These interactions—both among stromal cells (e.g., pericytes, fibroblasts, smooth muscle cells) and between stromal and glial cells and stromal and immune cells —are critical for tissue homeostasis, including regulation of barrier integrity, nutrient delivery, and immune surveillance. Disruption of their stoichiometry (e.g., altered pericyte-to-endothelial cell ratios) or function may contribute to disease progression.

Note 2: Additional proteins associated with T2LL

Proteins positively correlating with CEL# and T2LL were released by B cells (e.g., CD40, FAIM3), T cells (CD8A, LAG3, CRTAM), NK cells (GZMA, CD7, CST7) and monocyte/macrophages (e.g., TREM1, OLR1, TNFRSF1B, MMP9, CHIT1, CCL17) (Extended Figure 6a).

Development, quantity, and proliferation of neurons were decreased in pwMS with high T2LL in IPA analyses (z scores -3.39 to -2.77; p=8.06e-46 to 1.83e-22), as were intercellular junctions proteins (z scores -3.121 to -2.6; p= 1.65e-18 to 1.34e-17). These include epithelial junctions and junctions between myelin and axons. Thus, downmodulated oligodendrocyte proteins localize to the Node of Ranvier (SPOCK1, NFASC; p=0.049), paranodal junctions (JAM3, NFASC; p=0.0168) and axo-glial junctions (e.g., ALCAM, UNC5C, EPHB2, SEMA6A, NEO1; p=0.0024).

25% (17/69) of ExNeuron proteins decreased in patients with high T2LL were also globally decreased in MS CSF and included synaptic proteins (NRG1, NPTX1, ADAM23, LRRTM4, PCSK1, RTN4R, LRNF2 and PLCB1; p=0.007), enriched for glutamatergic synapse (p=8.19e-7) and proteins of ErbB signaling pathway

(p=0.00014). InhNeuron enriched proteins also highlighted synaptic loss in patients with high T2LL, specific for AMPA (VWC2, VWC2L, GRIA4; p=0.006) and GABA-ergic synapses (SV2A, CBLN4, NRXN3, CNTN5; p=0.006). Interestingly, these downmodulated proteins were linked to depression phenotype (p=0.0033; https://monarchinitiative.org/), mathematical ability (p=0.0031) and BMI (p=0.0016). We confirmed that 65% of these InhNeuron proteins were significantly downmodulated in obese pwMS (Figure 9b, Supplementary data 3).

Astrocyte proteins downmodulated in pwMS with high T2LL were enriched for plasma membrane receptor complexes and secreted proteins. As only 19% of these astrocyte proteins were decreased in MS CSF compared to HV (e.g., BMPR1A, IL20RA, WIF1, SPON1), downmodulation of astrocytic proteins in people with high T2LL more likely reflects increased consumption/internalization of these proteins in response to CNS injury than astrocyte loss. This conclusion is supported by proteins secreted by astrocytes that we found increased in people with high T2LL but not globally different between MS and HV: ANGPT1 and TNC that support VEC- and neuronal regeneration, respectively. Only astrocytic STOX1 and SERPINA3 correlated positively with T2LL and were increased in MS CSF: STOX1 regulates reactive oxygen species (ROS), while SERPINA3, physiologically expressed in fibroblasts, is pathologically released by neurotoxic astrocytes[1] and was previously linked to MS severity[2].

From 371 proteins correlating with T2LL but not CEL# (and therefore likely representing evolving MS mechanisms), 156 (42%) correlated positively with T2LL. Of these, the X-chromosome linked AIFM1, mitochondrial NADH oxidoreductase that maintains electron transport but also mediates caspase-independent apoptosis, showed strongest effect size. AIFM1 mutations cause hypomyelination[3] and we found AIFM1 enriched in MS CSF (p=5.21e-20), in females more than males (p=7.84e-3). AIFM1 increase in MS females suggests possibility of partial escape from X-chromosome inactivation[4] (Supplementary Fig. 3c-e). AIFM1 is also highly expressed in EBV-infected B cells and exploited by viruses because it sensitizes T cells to activation-induced apoptosis[5]. Thus, AIFM1 exemplifies X-linked protein that may play dual role in MS by affecting both anti-viral/EBV immunity and resistance of CNS cells to stress-induced apoptosis.

Note 3: Proteins linked to progression of cognitive disability, extended results

The myeloid cell proteins involved in cell surface interactions at the vascular wall that positively correlate with CEL# and BD include TREM1, MMP8, ORL1, CD84, SELPG.

Seven macromolecular functions positively (i.e., z-score>2) correlated with BD: 3 were linked to connective tissue cells (quantity of connective tissue [p=5.17e-20], quantity of connective tissue cells [p=2.17e-15], development of connective tissue cells [p=2.24e-15]), 2 to osteoclasts (myeloid cells derived, participating in ECM remodeling; osteoclastogenesis [p=8.46e-16], quantity of osteoclasts [p=2.24e-15]), and remaining 2 to vaso-occlusion (vaso-oclusion [p=1.44e-22], occlusion of artery [p=4.41e-20]).

64% of proteins correlating with BD differed between sexes (Figures 2c and 4c). Males had increased levels of 73% of proteins positively correlating with BD. These likely pathogenic proteins participated in matrix metalloproteinases (MMP) activation (p=0.0223), ECM organization (p=0.00027), collagen degradation (p=0.0031), formation of fibrin clot (p=4.68e-6), platelet activation (6.85e-5) and complement regulation (p=0.0085). Conversely, 66% of proteins negatively correlating with BD and

exerting sex effect were increased in female pwMS. These were involved in generation of neurons (p=7.72e-11), axon guidance (p=2.23e-10; exemplified by OLFM1 in Figure 6c) and Notch signaling (p=0.0248).

46 biomarkers (Supplementary Fig. 6c) and 3 pathways (Hepatic fibrosis, LXR/RXR activation and HIF1a signaling) correlated positively with BD, were upregulated in pwMS with disproportionally high BD and downmodulated in patients with disproportionally low BD. Out of 23 cell-specific proteins 39% originated from stromal cells and 17% from myeloid cells. Consistently, IPA linked myeloid cells/phagocytosis (z scores 4.037 to 2.77, p=1.31e-26 to 1.6e-13) and fibroblasts/connective tissue functions (z scores 3.48 to 2.905; p=5.6e-31 to 3.44e-18), but also viral infections (z-scores 3.156 to 2.285; p=6.44e-35 to 1.67e-23) to disproportionally high BD (Supplementary Fig. 6d).

Note 4: Shared mechanisms linked to development of cognitive and physical disability (i.e., correlating with Global MS Disability outcome; GMDS) extended results

Pathways with strongest positive GMSD correlations included IL-6 signaling, TNFs bind their physiological receptors, Role of Macrophages, Fibroblasts, Endothelial Cells and Osteoblasts, Osteoclasts and Chondrocytes in Rheumatoid Arthritis, Neutrophil degranulation, Production of NO and ROS in Macrophages, but also HMGB1 signaling and Immunogenic Cell Death Signaling (Figure 7c). To exclude spurious negative correlations with GMSD, we filtered-out pathways positively correlating with CEL#; remaining candidate protective pathways included RAF/MAP kinase cascade (important in tissue repair, cell survival and neuronal development), ROBO-SLIT signaling (involved in axon guidance and angiogenesis), Clathrin-mediated Endocytosis Signaling, IL13 signaling, IL15 production and others.

Proteins negatively correlated with GMSD and decreased in MS CSF included neuronal proteins CHGB, BDNF, CDHR1, LRRTM4 and LRRC37A2 and ROBO2. As these proteins were transcriptionally decreased in chronic active MS lesions without transcriptional downregulation in individual neurons (Supplementary data 3), their CSF decrease reflects neuronal loss. Likewise, multiple oligodendroglial, OPC and VEC enriched proteins correlated with GMSD without their transcriptional downregulation, reflecting loss of these cells.

Note 5: Molecular mechanisms associated with development of physical disability and SC injury overlap with mechanisms of brain non-lesional activity (i.e., development of brain atrophy out of proportion to T2LL). Extended results.

Among 63 proteins/pathways positively associated with SC disability (based on positive correlations with SC disability and enrichment in appropriate propensity-matched groups) were components of NFkB signaling pathway (p=0.0017), and ECM (p=0.0079), including collagen trimers (p=0.03). The pathways preferentially linked to SC injury were Hepatic fibrosis, STAT3 signaling, HIF1a, Collagen degradation, Inhibition of Matrix Metalloproteases, Role of PKR in Interferon Induction and Antiviral Response.

Note 6: Biomarkers changing with CombiWISE disability progression rates in longitudinal cohort. Extended results.

54% of proteins changing congruently with CombiWISE disability progression slopes were cell-enriched (8 fibroblast-, 5 LEC- and 5 Schwann cells-enriched). These were enriched for ECM proteins (p=0.0298), VEGFA-KDR signaling components (p=0.047), important for lymphangiogenesis and angiogenesis alike. Consistently, activation of pathways linked to inflammation-mediated tissue remodeling by stromal cells reflected increase in disability progression (Figure 8i).

Only yearly change in 2 proteins (B cell enriched FCRL3 and granulocyte secreted ADCYAP1) and 6 pathways correlated negatively with disability progression slopes. ADCYAP1 mediates neuroendocrine stress response that promotes development of neuron projections. FCRL3 is intracellular protein preferentially expressed in B cells, but also some NK and T cells. Even though B-, T- and NK cell markers are generally increased in MS CSF, FCRL3 is not, suggesting that B cells may release FCRL3 into CSF only during immunogenic cell death.

The yearly change in pathways negatively correlating with rates of disability progression include activation of nuclear receptors (RAR-, FXR/RXR) that promote remyelination[6,7] but, surprisingly, also immune pathway (Crosstalk between DC and NK cells) that may be important for control of viral infections.

Note 7: Compared to Caucasian MS patients, African American (AA) participants show stronger intrathecal inflammation and more profound CNS injury

As we did not have enough participants to investigate effects of all races, we focused on differences between Caucasian and African American (AA) pwMS representing 18.9% of our MS cohort.

CSF levels of 1439 proteins and 118 pathways differed between Caucasian and AA pwMS after adjusting for sex and age effects (Supplementary data 3). 575 (40%) of race-divergent proteins were elevated in AA pwMS. These were mostly immune cell-related proteins participating in NFkB signaling (p=1.91e-7) and linked to viral infections, including EBV (p=0.0015; MAVS, FCER2, CD40, TABP, HLA-DMA, HLA-DPB1, ICAM1, TNF, STAT1, JUN, OAS1, CDKN1B). 74/118 (63%) of race-divergent pathways were elevated in AA pwMS and, consistent with pro-inflammatory skewing of proteins, these were dominated by pro-inflammatory pathways, such as Natural Killer Cell Signaling, CGAS-STING signaling, Neuroinflammation Signaling, Neutrophil Extracellular Trap Signaling.

In contrast, 864 proteins decreased in AA pwMS were enriched for CNS cell-specific proteins. Indeed, proteins derived from ExNeurons, InhNeurons, oligodendrocytes, OPCs, astrocytes, VEC, LEC and microglia were preferentially decreased in CSF of AA pwMS (Figure 3). Consistently, 44 pathways significantly downmodulated in AA pwMS included Myelination signaling, Synaptogenesis, Axonal guidance signaling, but also Collagen degradation, IL-10 signaling, HIF1a signaling, and IL27 signaling.

These results implicate strong intrathecal inflammation and higher CNS injury in AA pwMS as the main race effect and are consistent with more relapses and faster accumulation of disability observed in AA pwMS[8].

Note 8: CSF proteome of MS patients with smoking history reflects increased activation of cytotoxic cells and decreased Netrin-1 signaling

Although smoking, including history of passive smoke exposure increases MS incidence and may also influence MS outcomes, how smoking mediates these effects is unknown. We found 214 CSF proteins significantly associated with smoking history after age/sex adjustments (Supplementary data 3). 61% (131/214) of these were increased in current or past smokers; among these were cytotoxic T cells and NK cells proteins CD8A, LAG3, CRTAM, SIRPG, GZMM, CST7 and RUNX3, although this cell-specific enrichment did not reach formal statistical significance. Interestingly, markers enriched in naïve B cells (FCRL1, IgD) were also increased in CSF of past/current smokers. 83 CSF proteins decreased in smokers included immunoregulatory PDCD1, IL7 (secreted by stromal cells and consumed by T/NK cells), oligodendroglial CNP and OPC-enriched NTN4. Netrin-1 signaling, that guides migration of neurons and their axonal growth was the only pathway linked to smoking, and it was downmodulated in smokers.

Note 9: BMI affects CSF biomarkers of lipid metabolism, complement activation and neuronal biology, while obesity increases intrathecal inflammation and decreases neuronal physiological processes such as synaptogenesis

Obesity (during childhood and adolescence) increases risk of MS and may also accelerate rates of disability progression[9]. To identify how obesity affects MS, we modeled the effect of BMI on CSF biomarkers as a continuous or categorical variables (normal [BMI<30] vs obese [BMI=>30]), adjusting for sex and age effects (Figure 9a, Supplementary data 3). Due to overlapping results, we'll describe continuous analysis.

From 937 proteins significantly associated with BMI only 167 (18%) correlated positively with BMI (Figure 3). Reassuringly, the highest effect sizes exerted proteins linked to obesity such as LEP, FABP3, FABP4 and CRP (Figure 9b-c). Additionally, components of complement (p=1.89e-14), including those constituting membrane attack complex (C5, C6, C8A, C9) and coagulation cascade (p=0.00044) were enriched among proteins positively correlating with BMI. Consistently, among 45 IPA pathways positively correlating with BMI, pathways linked to lipid metabolism (RAR activation, FXR/RXR activation) and coagulation (Hematoma Resolution Signaling Pathway) exerted high effect sizes. In contrast, we observed preferential negative correlations with BMI for many cell-specific proteins (Figure 3, Supplementary data 4). This included proteins derived from CNS cells: ExNeurons, InhNeurons, oligodendrocytes, OPCs, Schwann cells and astrocytes (Figure 3). As many of these proteins also negatively correlated with disability outcomes and were generally decreased in pwMS, their negative correlation with BMI indicates greater loss of the CNS cells, or their physiological functions in obese pwMS. These essential CNS proteins are exemplified by neuronal pentraxin NPTX1 and myelin component CNP. Surprisingly, myeloid cell proteins (i.e., from monocytes, microglia and granulocytes), LEC proteins and PB/PC proteins were also skewed towards negative associations with BMI, suggesting that obesity also limits some immune responses. Consistently, IPA pathways related to myeloid cells (e.g., IL10, IL6 signaling, Granulocyte adhesions and diapedesis, Role of Osteoclasts in Rheumatoid Arthritis Signaling Pathway) correlated negatively with BMI.

We conclude that intrathecal effects of obesity overlap with its systemic effects and are characterized by dysfunction of lipid metabolism, but also with decreased concentration of CSF proteins originating from myeloid cells, which are generally positively associated with MS progression and accumulation of disability. Despite this poor recruitment of myeloid cells or their lack of activation, obesity is linked to greater CNS cell injury, consistent with its known detrimental effect on disability accumulation.

Note 10: DRB1*15:01 genotype increases plasmablasts/plasma cell (PB/PC) proliferation and IgG secretion, but may also protect patients from MS-associated neuronal loss

Although HLA-DRB1*15:01 in Caucasians and homologous allele (DRB1*15:03) in AA represent the strongest genetic risk factors for MS, surprisingly little is known about how this genotype affects intrathecal molecular disease mechanisms. We thus again performed 2 analyses: for DRB1*15:01 only and for DRB1*15:01+DRB1*15:03 (Supplementary Table 4).

Constructing sex- and age-adjusted linear model from MS patients who are homozygous, heterozygous or do not express HLA-DRB1*15:01, we found 1478 CSF biomarkers and 164 pathways significantly associated with DRB1*15:01 haplotype and 1184 proteins/83 pathways associated with DRB1*15:01/03 haplotype (Figure 9d). 94% (1185/1267) of DRB1*15:01/03 haplotype-linked proteins were also significantly associated with DRB1*15:01 haplotype with identical directionality and effect sizes (coefficient R^2=0.9816, p<0.0001). As proteins linked solely to DRB1*15:01 haplotype were enriched for proteins differentially expressed between Caucasian and AA pwMS, we focused on combined analysis of DRB1*15:01/03 to discern the effect of this MHC-II association with MS.

As DRB1*15:01/03 haplotype is linked to MS incidence but not MS severity, we expected to detect its influence on immune cells. Indeed, we observed significant positive association of proteins derived from PB/PC, but not other immune cells with DRB1*15:01/03 haplotype (Figure 3). Surprisingly, this haplotype was also associated with significant enrichment of most CNS cell- specific proteins, including ExNeurons, InhNeurons, Oligodendrocytes, OPC, astrocytes, VEC and microglia (Figure 9d-e). This was unexpected, but consistent with IPA predicting DRB1*15:01/03 haplotype-associated with decreased Apoptosis of neurons (z=-3.599, p=4.13e-19), increased Development of neurons (z=2.284, p=6.33e-48), increased Proliferation of neural cells (z=2.225, p=1.23e-28), increased Developmental process of synapse (z=2.774, p=2.20e-20) and increased Assembly of intercellular junctions (z=2.502, p=8.06e-22), including Development of gap junctions (z=2.413, p=3.74e-22). These processes either limit CNS injury or enhance its repair, explaining lack of association of DRB1*15:01/03 haplotype with MS severity. On the side of the immune system IPA predicted increases in lymphopoiesis (z=2.975, p=4.15e-25), viral infection (z=2.341, p=4.85e-32), T cell development (z=2.462, p=1.15e-20) and phagocytosis (z=2.229, p=1.29e-18) (Figure 9f).

We conclude that CSF proteome linked to DRB1*15:01/03 haplotype reflects activation of immune responses that may be part of antiviral immunity. This DRB1*15:01/03-associated inflammation seems to permit or induce environment that limits CNS injury and mediates effective CNS repair.

Note 11: Unified hypothesis that integrates CSF proteomic results.

Is there a unifying explanation for presented results? If we were to speculate, we would suggest that our results are consistent with persistent EBV infection, brought intrathecally by latently infected B cells and associated with occasional lytic reactivations that damage CNS epithelial barriers. This likely leads to activation of anti-viral immunity and tissue remodeling. Indeed, EBV can infect human VEC[10], including those of brain[11]. Infection of epithelial cells not only impairs barrier function but also promotes EBV reactivation[12], potentially constituting both the initial insult to the BBB and the source of chemokines and adhesion molecules that facilitate immune cell recruitment to perivascular spaces—hallmarks of contrast-enhancing lesion (CEL) formation.

But does this activation/recruitment of immune cells mediate subsequent CNS injury? We have not found expected correlation between activation of T cell immunity and CEL-associated axonal damage. Instead, CEL destructiveness correlated with the extent of BBB disruption, extracellular matrix (ECM) remodeling, and fibroblast activation. These findings are consistent with the histopathological observation of spindle-shaped demyelinated lesions centered on a central venule, implicating diffusion of soluble factor(s), rather than cell-mediated cytotoxicity, as the primary mechanism of oligodendroglial injury, demyelination, and axonal transection.

If BBB damage is indeed triggered by lytic EBV reactivation, then, consistent with our results, effective cellular immunity that restricts lytic replication may be neuroprotective. This would reconcile the observation that T cell activation is linked to the presence of CELs, but not to their destructiveness.

We further hypothesize that the activation of stromal cells, leading to secretion of ECM components, represents an initially beneficial compensatory response aimed at limiting CEL destructiveness. Even the phenotypic transformation of epithelial cells, particularly lymphatic endothelial cells (LECs), and stromal cells such as pericytes and smooth muscle cells, in response to barrier injury, may reflect an adaptive tissue remodeling program. This process, including the formation of tertiary lymphoid follicles (TLF), may serve to preserve tissue integrity under conditions of persistent viral infection by supporting local antigen presentation and promoting the differentiation and survival of antiviral adaptive immune cells within CNS tissue.

However, by supporting the intrathecal survival of B cells and their differentiation into plasmablasts and plasma cells (PB/PC), these same stromal elements may inadvertently sustain a chronic intrathecal reservoir of EBV, perpetuating a cycle of latent infection and episodic reactivation.

The striking expansion of fibroblast-derived proteins and their preferential positive correlation with the accumulation of cognitive and physical disability, independent of new MS lesion formation, suggests that excessive tissue remodeling may convert an initially protective response into a maladaptive fibrotic process. A mechanism originally intended to limit the diffusion of harmful soluble factors into CNS tissue, which requires strict osmotic and ionic homeostasis, may ultimately impede the diffusion of essential nutrients. This could lead to localized hypoxia, as evidenced by the upregulation of the HIF1α pathway[13].

However, if EBV persists and periodically reactivates in the CNS of pwMS, why is there no clear evidence for intrathecal production/enrichment of anti-EBV antibodies? This remains the weakest point of the continuous intrathecal EBV infection hypothesis. One possibility is that anti-EBV antibodies are rapidly cleared from CSF via immune complex (IC) formation. We present indirect evidence for ongoing IC formation in MS CSF. Although several studies reported IgG-containing ICs in the CSF of pwMS more

than two decades ago[14-18], this line of investigation should be revisited using modern technologies. Given the enrichment of myeloid phagocytes and NK cells in MS CNS tissue, it is plausible that ICs are preferentially phagocytosed, and their presence in CSF may reflect only the overflow. In contrast, multiple studies have consistently demonstrated enrichment of anti-EBV T cells in MS intrathecal compartment[19-21], supporting the hypothesis of ongoing EBV infection within CNS tissue.

Note 12: Orthogonal validation of the Somascan assay

Published studies:

1. https://somalogic.com/specificity : >5,000 SOMAmer reagents have at least one form of orthogonal confirmation, such as mass spectrometry or ELISA, with approximately 3,000 undergoing two or more validation methods
2. Our own 2017 study [22] using CSF Somascan-measured proteins as diagnostic test of MS and its progressive stage. In this study we addressed: (a) Technical assay variability (i.e. same CSF samples measured twice, on same or different plates and on same/different assay runs); (b) Biological variability: 2 different CSF samples collected from same (healthy) individuals on different occasions; (c) Somascan relative fluorescence units (RFUs) versus absolute protein concentrations measured by ELISA. We found that Somascan assay has excellent technical and biological reproducibility and majority of orthogonally-investigated CSF protein RFUs correlated moderately or strongly with absolute concentrations measured by ELISA assays and standard curves.
3. This 2018 study [23] extends our 2017 publication by demonstrating <20% coefficient of variation for 99% of Somamers, with >0.75 ICC for 61% of measured serum proteins.
4. This 2018 study [24] identified 502 cis pQTLs with Somamers – by definition of cis pQTL, these 502 Somamers must measure the targeted protein.
5. This 2024 study [25] analyzed CSF proteins in 300 participants using both Somascan assay and tandem mass tag mass spectrometry (TMT-MS). Their Table 4 lists 1274 CSF proteins measured by both modalities. Of these, 1168 (92%) showed statistically significant correlations with average Spearman Rho= 0.57 (Median 0.61, Max 0.89, Min 0.2)

Supplementary notes references:

1. Liddelow, S.A., et al. Neurotoxic reactive astrocytes are induced by activated microglia. Nature (2017).
2. Masvekar, R., Kosa, P., Barbour, C., Milstein, J.L. & Bielekova, B. Drug library screen identifies inhibitors of toxic astrogliosis. Multiple sclerosis and related disorders 58, 103499 (2022).
3. Edgerley, K., et al. AIFM1-associated X-linked spondylometaphyseal dysplasia with cerebral hypomyelination. Am J Med Genet A 185, 1228–1235 (2021).
4. Balaton, B.P., Cotton, A.M. & Brown, C.J. Derivation of consensus inactivation status for X-linked genes from genome-wide studies. Biol Sex Differ 6, 35 (2015).
5. Ji, W., et al. Newly synthesized AIFM1 determines the hypersensitivity of T lymphocytes to STING activation-induced cell apoptosis. Cell reports 42, 112327 (2023).
6. Natrajan, M.S., et al. Retinoid X receptor activation reverses age-related deficiencies in myelin debris phagocytosis and remyelination. Brain 138, 3581–3597 (2015).
7. Huang, J.K., et al. Retinoid X receptor gamma signaling accelerates CNS remyelination. Nat Neurosci 14, 45–53 (2011).
8. Khan, O., et al. Multiple sclerosis in US minority populations: Clinical practice insights. Neurol Clin Pract 5, 132–142 (2015).
9. Lutfullin, I., et al. Association of obesity with disease outcome in multiple sclerosis. J Neurol Neurosurg Psychiatry (2022).
10. Jones, K., et al. Infection of human endothelial cells with Epstein-Barr virus. J Exp Med 182, 1213–1221 (1995).
11. Casiraghi, C., Dorovini-Zis, K. & Horwitz, M.S. Epstein-Barr virus infection of human brain microvessel endothelial cells: a novel role in multiple sclerosis. J Neuroimmunol 230, 173–177 (2011).
12. Temple, R.M., et al. Efficient replication of Epstein-Barr virus in stratified epithelium in vitro. Proc Natl Acad Sci U S A 111, 16544–16549 (2014).
13. Halder, S.K. & Milner, R. Hypoxia in multiple sclerosis; is it the chicken or the egg? Brain 144, 402–410 (2021).
14. Jans, H., et al. Immune complexes and the complement factors C4 and C3 in cerebrospinal fluid and serum from patients with chronic progressive multiple sclerosis. Acta Neurol Scand 69, 34–38 (1984).
15. Procaccia, S., et al. Circulating immune complexes in serum and in cerebrospinal fluid of patients with multiple sclerosis. Characterization and correlation with the clinical course. Acta Neurol Scand 77, 373–381 (1988).
16. Salmi, A., Ziola, B., Reunanen, M., Julkunen, I. & Wager, O. Immune complexes in serum and cerebrospinal fluid of multiple sclerosis patients and patients with other neurological diseases. Acta Neurol Scand 66, 1–15 (1982).
17. Sindic, C.J., Cambiaso, C.L., Depre, A., Laterre, E.C. & Masson, P.L. Immune complexes in cerebrospinal fluid and serum of neurological patients. Possible intrathecal formation in bacterial meningitis and herpetic encephalitis. J Neuroimmunol 6, 9–18 (1984).
18. Coyle, P.K. CSF immune complexes in multiple sclerosis. Neurology 35, 429–432 (1985).
19. Jaquiery, E., et al. Intrathecal immune responses to EBV in early MS. Eur J Immunol 40, 878–887 (2010).
20. Jilek, S., et al. Strong EBV-specific CD8+ T-cell response in patients with early multiple sclerosis. Brain 131, 1712–1721 (2008).
21. Lossius, A., et al. High-throughput sequencing of TCR repertoires in multiple sclerosis reveals intrathecal enrichment of EBV-reactive CD8+ T cells. Eur J Immunol 44, 3439–3452 (2014).

22. Barbour, C., et al. Molecular-based diagnosis of multiple sclerosis and its progressive stage. Ann Neurol 82, 795–812 (2017).
23. Kim, C.H., et al. Stability and reproducibility of proteomic profiles measured with an aptamer-based platform. Sci Rep 8, 8382 (2018).
24. Sun, B.B., et al. Genomic atlas of the human plasma proteome. Nature 558, 73–79 (2018).
25. Dammer, E.B., et al. Proteomic analysis of Alzheimer's disease cerebrospinal fluid reveals alterations associated with APOE epsilon4 and atomoxetine treatment. Sci Transl Med 16, eadn3504 (2024).

Supplementary Tables

Supplementary Table 1: Demographic data

	all samples				untreated samples only		
	**HC**	**RRMS**	**PPMS**	**SPMS**	**RRMS**	**PPMS**	**SPMS**
Number of samples	69	345	375	251	162	321	151
Number of participants	44	179	124	91	143	112	74
Percentage of females (%)	47.7	64.8	49.2	62.6	64.3	47.3	62.2
Age at 1st LP							
Min - Max (years)	19.4 - 71.3	18.0 - 68.6	25.3 - 74.7	22.0 - 69.6	18.0 - 68.6	25.3 - 70.4	22.0 - 69.6
Mean ± SD (years)	37.8 ± 13.4	39.4 ± 10.5	54.2 ± 9.2	51.3 ± 10.3	38.7 ± 10.3	54.0 ± 9.3	51.5 ± 10.4
Disease duration at 1st LP							
Min - Max (years)	-	0.0 - 36.3	0.0 - 38.8	0.3 - 42.4	0.0 - 33.1	0.0 - 40.7	0.3 - 42.4
Median (IQR) (years)	-	1.9 (8.8)	9.2 (10.3)	20.2 (16.3)	1.3 (5.6)	10.0 (11.4)	20.4 (16.7)
EDSS at 1st LP							
Min - Max (years)	0.0 - 2.5	0.0 - 6.5	1.5 - 8.5	1.5 - 8.0	0.0 - 6.5	1.5 - 8.5	1.5 - 8.0
Median (IQR) (years)	0.0 (1.0)	1.5 (1.5)	6.0 (2.5)	6.0 (1.5)	1.5 (1.5)	6.0 (2.5)	6.5 (1.0)
Length of follow-up							
Min - Max (years)	0.0 - 4.7	0.0 - 19.8	0.0 - 9.2	0.0 - 16.8	0.0 - 8.6	0.0 - 9.2	0.0 - 16.8
Mean ± SD (years)	0.9 ± 1.4	2.3 ± 3.8	3.0 ± 2.6	2.3 ± 3.3	0.5 ± 1.7	2.7 ± 2.3	1.7 ± 2.8
Number of samples per subject							
Min - Max	1 - 4	1 - 8	1 - 8	1 - 7	1 - 3	1 - 6	1 - 7
Mean ± SD	1.6 ± 0.9	1.9 ± 1.4	3.0 ± 1.7	2.8 ± 1.8	1.1 ± 0.4	2.9 ± 1.5	2.0 ± 1.5
Percentage of samples treated	-	53.0	14.4	39.8	-	-	-

HC – healthy control, RRMS – relapsing-remitting multiple sclerosis (MS), PPMS – primary progressive MS, SPMS – secondary progressive MS, LP – lumbar puncture, Min – minimum, Max – maximum, IQR – intraquartile range, SD – standard deviation

Supplementary Figures

Supplementary figure 1

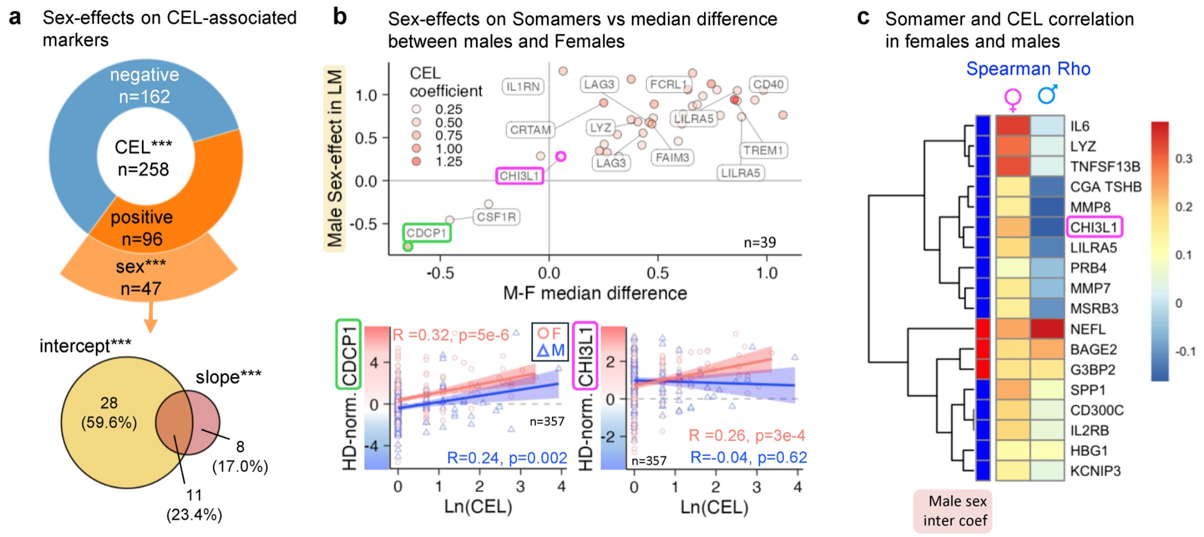

Supplementary Fig. 1: Residual sex effects in markers linked to numbers of contrast-enhancing lesions (CEL#) on brain magnetic resonance imaging (MRI) of people with multiple sclerosis (pwMS)

a, Number of somamers significantly associated with CEL#, with positive (blue) and negative (orange) regression coefficients. Among positively associated somamers, 48% also showed sex effects: significant intercept differences (yellow circle), significant slope differences (red circle), or both (overlay) in linear regression models of CEL#.

b, Scatter plot of somamers with significant sex effect on intercept. The x-axis shows male–female differences in somamer levels, and the y-axis shows the male sex coefficient (higher = stronger male association). Circle fill color corresponds to the CEL# coefficient in the regression model. Examples shown: CDCP1 (left) and CHI3L1 (right). Scatter plots depict correlations between CEL# (x-axis) and healthy control (HC)-normalized somamer levels (y-axis) in MS samples with CEL data. Female samples are shown as pink circles, male samples as blue triangles. Regression lines with 95% confidence intervals (CI) are plotted separately for females (pink) and males (blue).

c, Heatmap of proteins with significant CEL#:sex interactions in linear models. Heatmap color shows Spearman correlation coefficient (R^2) of CEL# with somamer levels in females (left, pink) and males (right, blue). Directionality of the CEL#:sex interaction coefficient is indicated: negative in males (blue) and positive in males (red).

All p values of regression coefficients were calculated using two-sided tests; displayed p values were not adjusted for multiple comparisons.

Supplementary figure 2

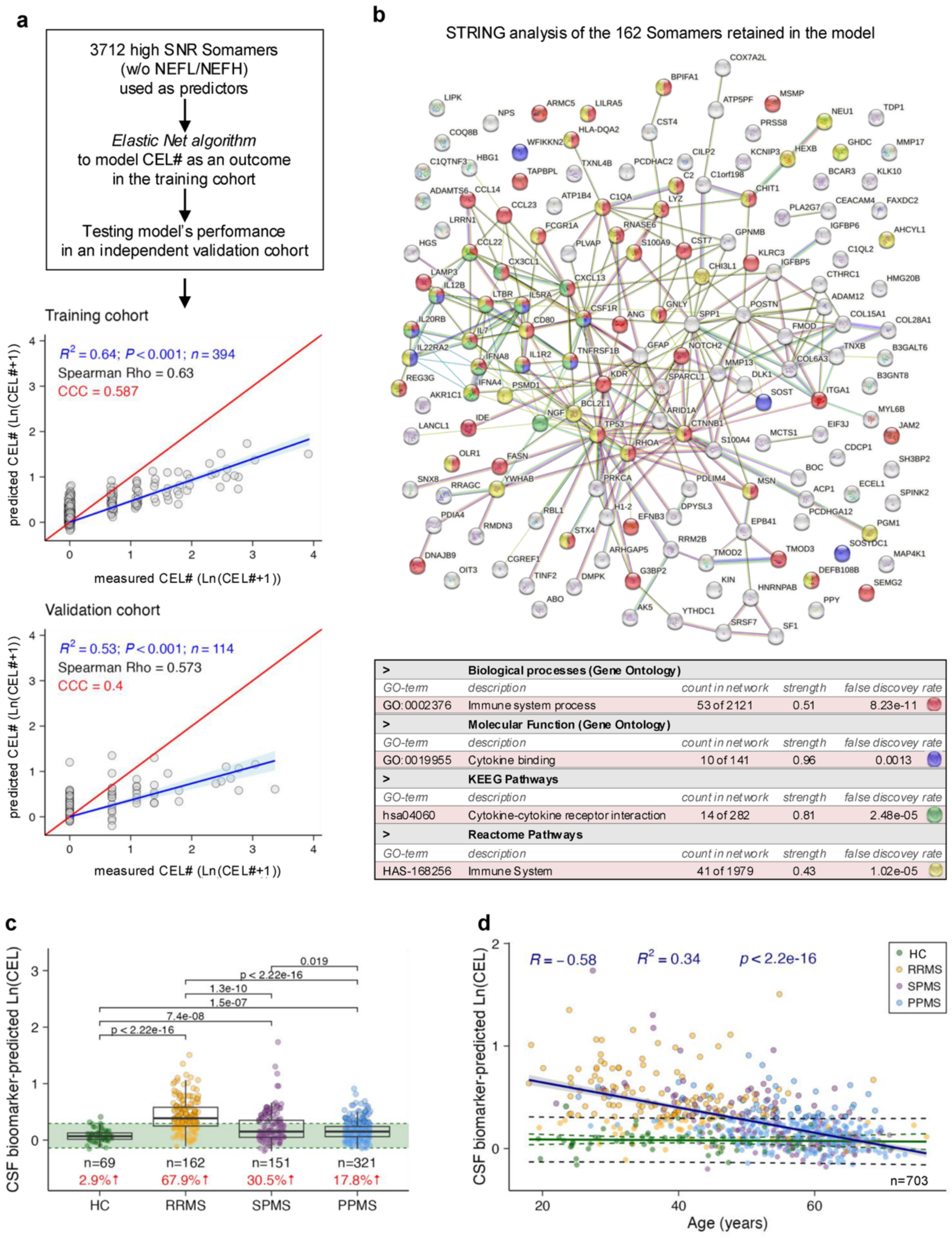

Supplementary Fig. 2: Cerebrospinal fluid (CSF) biomarker-based model of contrast-enhancing lesion number (CEL#) in people with multiple sclerosis (pwMS)

a, A total of 3,712 high signal-to-noise ratio (SNR) somamers (excluding neurofilament light [NEFL] and heavy [NEFH] chains) were used as predictors in an elastic net model of CEL#. The model was trained in one cohort (top) and validated in an independent cohort (bottom). Scatter plots show correlations between measured CEL# (x-axis, natural logarithm [Ln] of CEL#+1) and model-predicted CEL# (y-axis).

Evaluation metrics include coefficient of determination (R^2), p value, Spearman correlation coefficient (Rho), and Lin's concordance correlation coefficient (CCC). Blue line with 95% confidence interval (CI) shown; red line indicates 1:1 reference.

b, STRING analysis of the 162 somamers selected by the model identified enrichment in immune system processes, cytokine binding, cytokine–cytokine receptor interactions, and related immune functions. Categories are highlighted in red, blue, green, and yellow, respectively (see Supplementary data 11–12).

c, CSF biomarker-predicted CEL# significantly differentiated healthy controls (HC) from untreated MS samples, and relapsing-remitting MS (RRMS) from primary progressive MS (PPMS) and secondary progressive MS (SPMS). The green rectangle shows HC mean ±1.5 standard deviations (SD). Red percentages indicate proportions above the HC range. Significance was tested with one-way ANOVA and post-hoc two-sided t-tests. Boxplots display medians, quartiles, whiskers (1.5×IQR), and outliers.

d, Linear regression of CSF biomarker-predicted CEL# and age at collection (untreated stage) showed decreasing CEL# with increasing age. Blue line with gray shading shows regression with 95% CI; HC regression line, 95% CI, and 95% prediction interval shown in green/black. Metrics include Pearson correlation coefficient (R), R^2, and unadjusted p value.

All regression p values were tested in two-sided models.

Supplementary figure 3

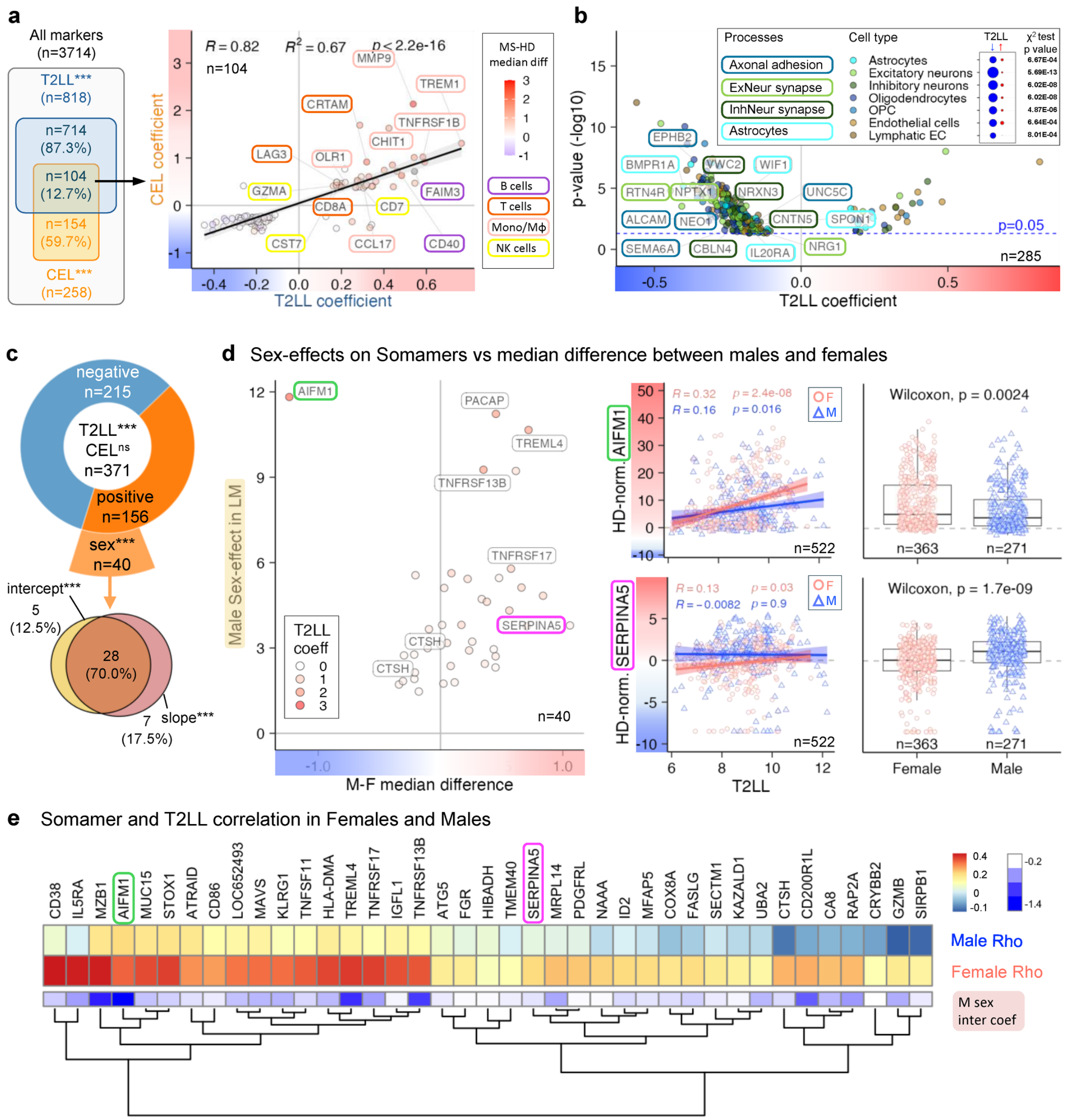

Supplementary Fig. 3: Cerebrospinal fluid (CSF) proteins associated with T2 lesion load (T2LL) measured on brain magnetic resonance imaging (MRI) of people with multiple sclerosis (pwMS)

a, Numbers of somamers significantly associated with contrast-enhancing lesions (CEL#), T2LL, and their overlap. Scatter plot of coefficients for somamers associated with both outcomes shows congruent directionality and strong correlation (black line with 95% confidence interval [CI], gray band), characterized by Pearson correlation coefficient (R), coefficient of determination (R^2), and unadjusted p value. Fill colors indicate median MS vs healthy control (HC) differences (red = elevated in MS; blue = decreased). Examples: B cell, T cell, monocyte/macrophage, and natural killer (NK) cell–specific biomarkers (purple, red, pink, yellow, respectively).

b, Volcano plot showing enrichment of astrocyte-, excitatory/inhibitory neuron-, oligodendrocyte-, oligodendroglial precursor cell (OPC)-, endothelial-, and lymphatic endothelial–derived proteins negatively associated with T2LL. One-sided chi-square tests identified significant enrichment. Ingenuity Pathway Analysis (IPA) highlighted molecules involved in axonal adhesion, synapse formation, and astrocyte activity.

c, Diagram showing numbers of somamers with significant sex associations among those positively associated with T2LL (orange) but not CEL#. Sex effects affected regression slopes (red circle) or intercepts (yellow circle).

d, Scatter plot of sex coefficients (y-axis) vs male–female somamer level differences (x-axis) shows male-elevated proteins often had higher intercepts in T2LL regression models. Examples: AIFM1 (green) and SERPINA5 (magenta). Regression lines with 95% CI are shown separately for females (pink) and males (blue), with Pearson R and unadjusted p values. Boxplots show sex differences in AIFM1 and SERPINA5, tested by Wilcoxon rank-sum test. Boxplots display medians, quartiles, whiskers (1.5×IQR), and outliers; gray dashed lines show HC means. Two CTSH labels correspond to distinct epitopes of the same protein.

e, Heatmap of somamers with T2LL:sex interactions affecting regression slopes. Fill color represents Spearman correlation between somamer and T2LL in males (blue, top) and females (pink, bottom). Tiles above dendrogram indicate male sex interaction coefficients.

All regression p values tested in two-sided models.

Supplementary figure 4

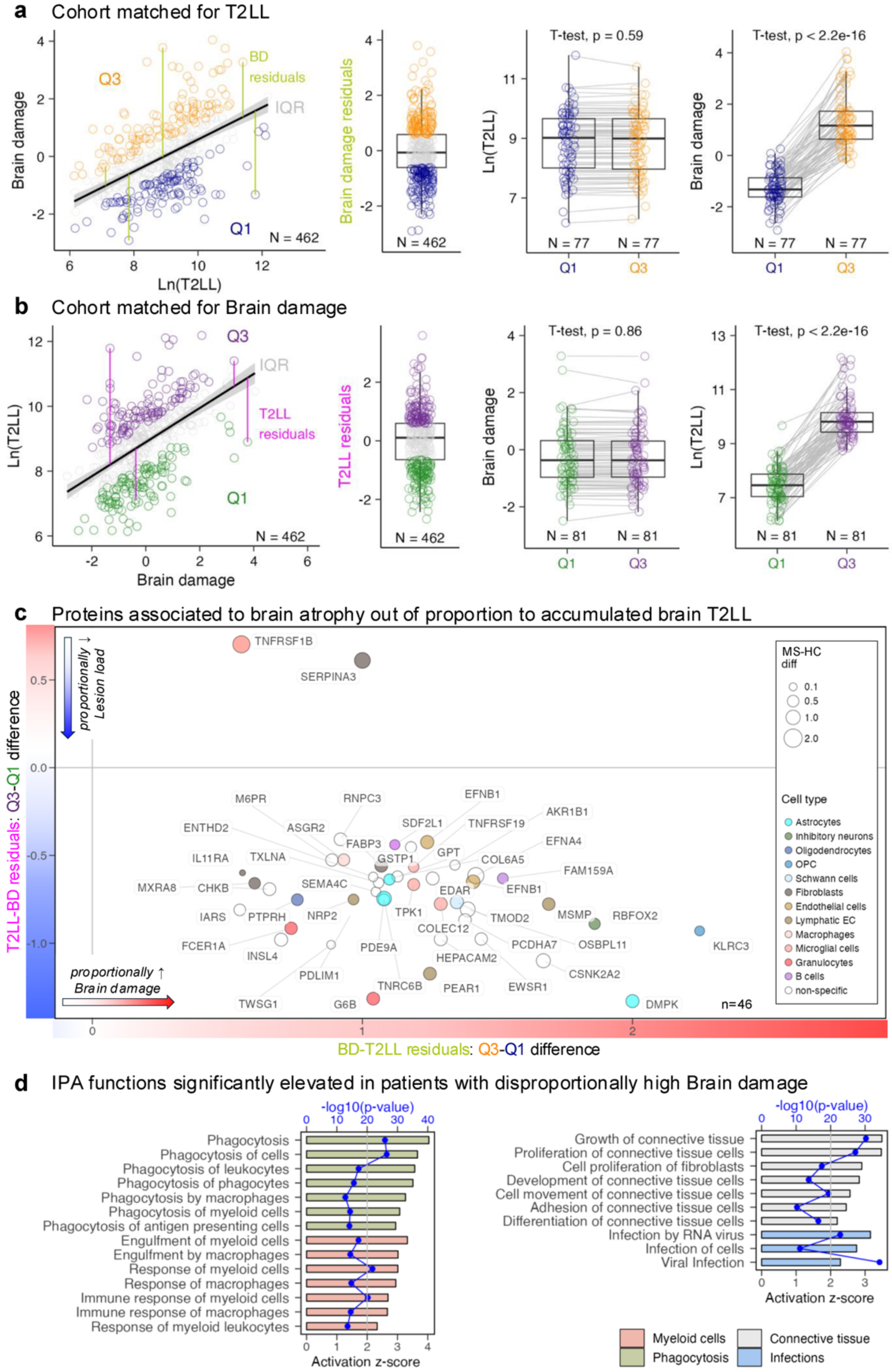

Supplementary Fig. 4: Cerebrospinal fluid (CSF) proteins and biological processes linked to multiple sclerosis (MS) non-lesional activity (i.e., accumulation of cognitive disability and brain atrophy out of proportion to T2 lesion load [T2LL])

a, Residuals (green vertical lines) from regression of brain damage (dependent) on T2LL (independent) were used to isolate two patient groups: Q1 (blue, residuals <25th percentile) and Q3 (yellow, residuals >75th percentile). Groups were propensity-matched to comparable T2LL but significantly different brain damage levels.

b, Similarly, residuals (magenta lines) from regression of T2LL (dependent) on brain damage (independent) identified Q1 (green, residuals <25th percentile) and Q3 (violet, residuals >75th percentile). Groups were matched for brain damage but differed in T2LL. Scatter plots show regression lines with 95% confidence intervals (CI, black/gray). Boxplots display medians, quartiles, whiskers (1.5×IQR), and outliers. Significance between Q1 and Q3 was assessed with paired t-tests (unadjusted p values).

c, Scatter plot of 46 proteins associated with disproportionate brain atrophy relative to T2LL. X-axis: Q3–Q1 difference in brain damage residuals (higher = proportionally greater brain damage); Y-axis: Q3–Q1 difference in T2LL residuals (lower = proportionally reduced lesion load). Circle size represents median somamer level differences between MS and healthy controls (HC); fill color denotes cell specificity.

d, Ingenuity Pathway Analysis (IPA) of somamers linked to disproportionate brain damage identified significantly elevated processes: myeloid cells (red), phagocytosis (green), connective tissue (gray), and infections (blue). Bar lengths represent activation z scores; blue dots indicate unadjusted pathway p values.

All regression p values were tested using two-sided models.

Supplementary Figure 5

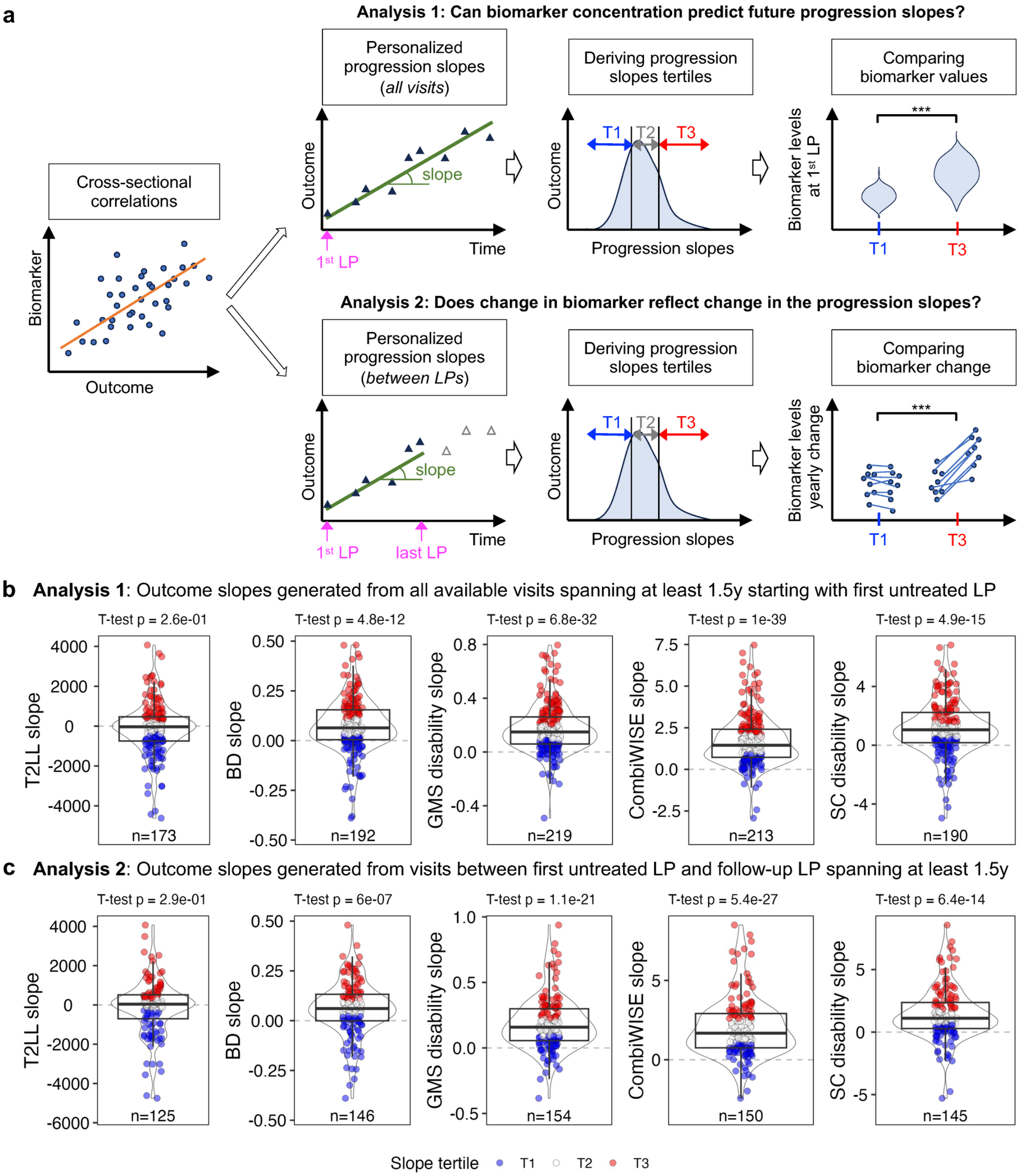

d Disability outcome slopes summary

	T2LL slopes				BD slopes				GMS disability slopes				CombiWISE slopes				SC disability slopes			
	Analysis 1		*Analysis 2*		*Analysis 1*		*Analysis 2*		*Analysis 1*		*Analysis 2*		*Analysis 1*		*Analysis 2*		*Analysis 1*		*Analysis 2*	
nr participants	*173*		*125*		*192*		*146*		*219*		*154*		*213*		*150*		*190*		*145*	
variable	f/u years	Nr visits	f/u years	Nr visits	f/u years	Nr visits	f/u years	Nr visits	f/u years	Nr visits	f/u years	Nr visits	f/u years	Nr visits	f/u years	Nr visits	f/u years	Nr visits	f/u years	Nr visits
Median	5.3	11	4.1	11	5.2	7	4.1	7	6.2	8	4.1	7	6.2	7	4.2	7	5.2	7	4.1	7
1st Qt.	3.6	7	3.2	8	3.8	4.8	3.1	5	4	5	3.1	5	4	5	3.2	5	3.9	4.2	3.2	5
3rd Qt.	8	16	6.2	15	7.3	10	5.9	9	9.8	11	6.1	9	9.9	11	6.1	9	7.4	10	5.9	9
Minimum	1.6	2	1.6	2	1.5	2	1.5	2	1.5	2	1.5	2	1.5	2	1.5	2	1.5	2	1.5	2
Maximum	16.7	47	13.4	42	12.1	18	9.7	14	20.1	36	13.5	31	20.1	36	13.5	31	12.4	18	9.7	14

Supplementary Fig. 5: Outcome progression slopes in multiple sclerosis (MS) longitudinal cohorts

a, Longitudinal analysis of cerebrospinal fluid (CSF) biomarkers was performed on somamers significantly associated with disability outcomes in cross-sectional cohorts. **Analysis 1** tested whether CSF biomarker levels at first lumbar puncture (LP, untreated stage) predicted future disability progression slopes. Progression slopes, calculated from clinic visits starting at the first LP, were divided into tertiles: slow (T1, blue), intermediate (T2, gray), and fast (T3, red). Biomarker levels at first LP were compared between T1 and T3 to identify predictors of disability accumulation. **Analysis 2** tested whether yearly biomarker change reflected progression slopes. Only visits between the first untreated LP and the last available CSF sample were included. CSF biomarker slopes were generated by regression of biomarker level vs age and compared between T1 and T3 groups.
b, Progression slopes for five disability outcomes—T2 lesion load (T2LL), brain damage (BD), global MS disability (GMSD), CombiWISE, and spinal cord (SC) disability—were calculated from visits beginning at the first untreated LP with ≥1.5 years of follow-up. Slopes were split into tertiles; slow vs fast progressors are shown in blue vs red. One-sample two-sided *t*-tests assessed whether average slopes differed from 0. Boxplots display medians, quartiles, whiskers (1.5×IQR), and outliers.
c, Progression slopes for the same five outcomes were also generated using only visits between the first untreated LP and follow-up LPs spanning ≥1.5 years.
d, Summary of progression slopes for all five disability outcomes from Analysis 1 and Analysis 2.

nr participants – number of participants, f/u – follow-up, Qt – quartile.

Supplementary figure 6

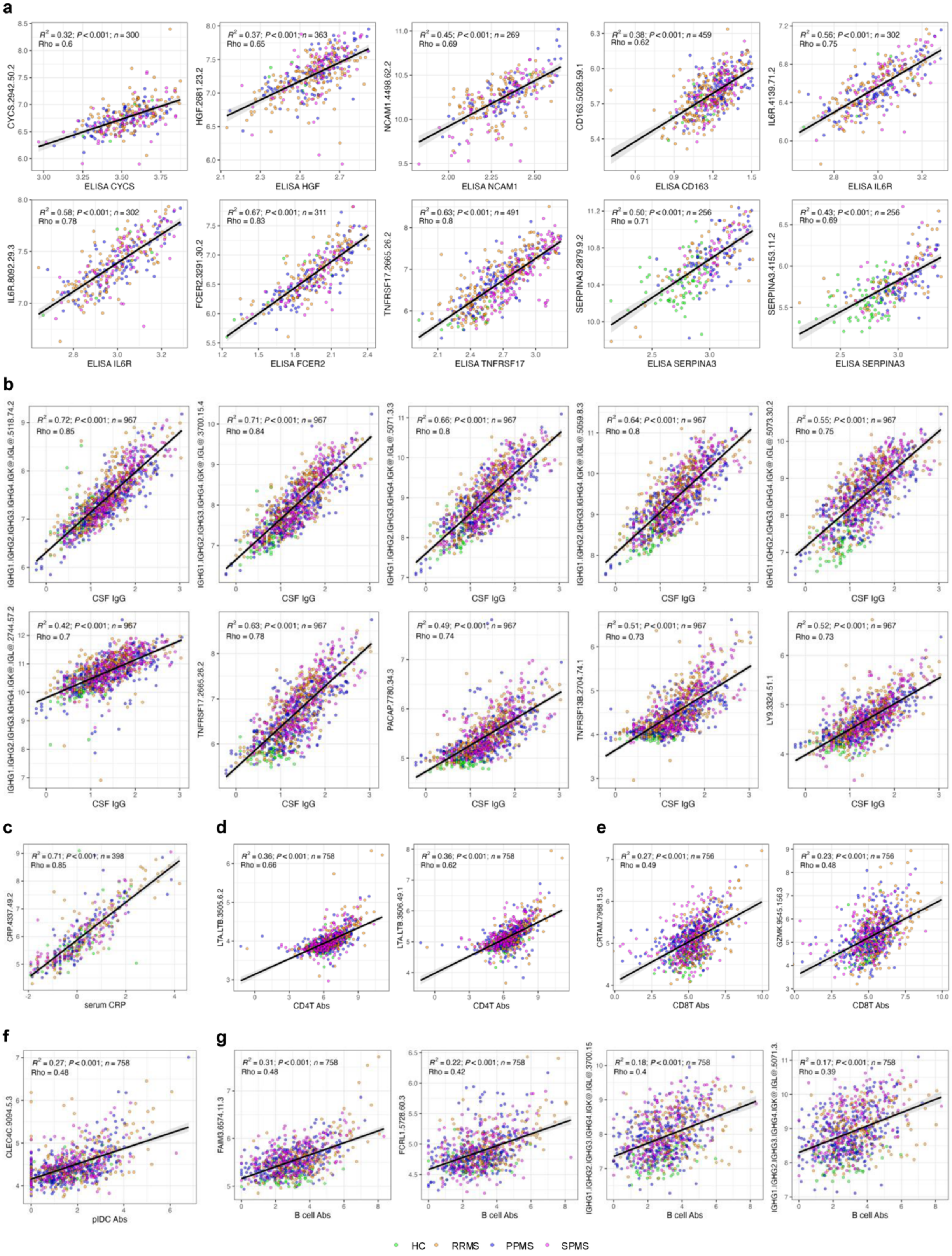

Supplementary Fig. 6: Orthogonal validation of SomaScan-based measurements of the SomaScan CSF biomarkers

a, Spearman correlations (Rho) between SomaScan-derived abundances and conventional ELISA concentrations for ten representative biomarkers; two independent somamers for IL-6R and SERPINA3 are shown. b, Correlations between CSF IgG concentrations determined by the NIH Clinical Center Department of Laboratory Medicine (DLM) and ten B-cell-associated somamers; six distinct somamers directed against IgG display comparable performance. c, Serum C-reactive protein (CRP) measured at the DLM versus CSF CRP quantified by SomaScan. d, Flow cytometry (FC)-derived absolute counts of $CD4^+$ T cells in CSF versus concentrations of T cell-secreted lymphotoxin-α/β (LTA/LTB) detected with two different somamers. e, FC $CD8^+$ T cell counts versus the T-cell markers CRTAM (class-I-restricted T-cell–associated molecule) and granzyme K (GZMK). f, FC absolute counts of CSF plasmacytoid dendritic cells (pDCs) versus the pDC-specific marker CLEC4C. g, FC absolute B cell counts versus three B cell-secreted proteins—FAIM3, FCRL1 and IGHG1—measured by SomaScan.

In all panels, the black line represents the least-squares regression with a grey 95 % confidence band; Spearman correlation coefficient (Rho), coefficient of determination (R^2), two-sided P value and sample size (n) are indicated. Both x and y axes values have been log-transformed (natural log). Samples originated from different diagnostic categories are color-coded: green circle for healthy controls (HC), orange for relapsing-remitting multiple sclerosis (RRMS), blue for primary progressive MS (PPMS) and pink for secondary progressive MS (SPMS).
