# Supplementary material for: Longitudinal proteomic profiling of cerebrospinal fluid in untreated multiple sclerosis defines evolving disease biology
Source: Nat Commun. 2025 Dec 3;16:11012. doi: 10.1038/s41467-025-65154-8 (PMC12695886; doi:10.1038/s41467-025-65154-8)
Supplement: Supplementary file 15 — Reporting Summary [file 41467_2025_65154_MOESM15_ESM.pdf]

nature portfolio

Corresponding author(s): Bibiana Bielekova

Last updated by author(s): Sep 18, 2025

Reporting Summary

Nature Portfolio wishes to improve the reproducibility of the work that we publish. This form provides structure for consistency and transparency in reporting. For further information on Nature Portfolio policies, see our Editorial Policies and the Editorial Policy Checklist.

Statistics

For all statistical analyses, confirm that the following items are present in the figure legend, table legend, main text, or Methods section.

n/a	Confirmed	
☐	☒	The exact sample size (*n*) for each experimental group/condition, given as a discrete number and unit of measurement
☐	☒	A statement on whether measurements were taken from distinct samples or whether the same sample was measured repeatedly
☐	☒	The statistical test(s) used AND whether they are one- or two-sided<br>*Only common tests should be described solely by name; describe more complex techniques in the Methods section.*
☐	☒	A description of all covariates tested
☐	☒	A description of any assumptions or corrections, such as tests of normality and adjustment for multiple comparisons
☐	☒	A full description of the statistical parameters including central tendency (e.g. means) or other basic estimates (e.g. regression coefficient) AND variation (e.g. standard deviation) or associated estimates of uncertainty (e.g. confidence intervals)
☐	☒	For null hypothesis testing, the test statistic (e.g. *F*, *t*, *r*) with confidence intervals, effect sizes, degrees of freedom and *P* value noted<br>*Give P values as exact values whenever suitable.*
☒	☐	For Bayesian analysis, information on the choice of priors and Markov chain Monte Carlo settings
☒	☐	For hierarchical and complex designs, identification of the appropriate level for tests and full reporting of outcomes
☐	☒	Estimates of effect sizes (e.g. Cohen's *d*, Pearson's *r*), indicating how they were calculated

Our web collection on statistics for biologists contains articles on many of the points above.

Software and code

Policy information about availability of computer code

Data collection	Clinical data was collected by board-certified neurologists and non-medical staff and recorded in our research database. MRI volumetric data was obtained from a commercial vendor Qmenta, Inc. (Boston, MA, USA). Somascan proteomic data was obtained from a custom 5k Somascan assay performed at the Somalogic, Inc (Boulder, CO, USA). NFL immunoassay was performed in-house using commercial reagents. We used commercial and publicly available data from the following databases - Ingenuity Pathway Analysis, (https://digitalinsights.qiagen.com/products-overview/discovery-insights-portfolio/analysis-and-visualization/qiagen-ipa/), Human protein atlas (https://www.proteinatlas.org), GTEx portal (https://gtexportal.org), STRING database (https://string-db.org), and Allen Brain Map (https://portal.brain-map.org). We used published snRNA-seq datasets (GSE180759, GSE118257, GSE227781 and PRJNA544731) and published datasets of scRNAseq of CSF cells (GSE133028, GSE163005, GSE138266, PRJNA866296, GSE172003, GSE277954). A newly generated single cell RNA seq dataset has been deposited to GEO database under accession number GSE286068.
Data analysis	All statistical analyses were performed using RStudio software Version 2023.12.1+402 (R version 4.3.3). The codes used to generate the data have been uploded to GitHub (https://github.com/Bielekova-Lab/Bielekova-Lab-Code/tree/master/FormerLabMembers/5k%20study%20R%20codes). For RNAseq analyses the following packages were used: Cellranger ver.7.0 (10x Genomics), Seurat (ver. 4.2.0), DoubletFinder V2.0, dsb package.

For manuscripts utilizing custom algorithms or software that are central to the research but not yet described in published literature, software must be made available to editors and reviewers. We strongly encourage code deposition in a community repository (e.g. GitHub). See the Nature Portfolio guidelines for submitting code & software for further information.

Data

Policy information about availability of data

All manuscripts must include a data availability statement. This statement should provide the following information, where applicable:

- Accession codes, unique identifiers, or web links for publicly available datasets
- A description of any restrictions on data availability
- For clinical datasets or third party data, please ensure that the statement adheres to our policy

Raw data is available in Supplementary Tables and in Github repository

Research involving human participants, their data, or biological material

Policy information about studies with human participants or human data. See also policy information about sex, gender (identity/presentation), and sexual orientation and race, ethnicity and racism.

Reporting on sex and gender	We have collected biological sex information on all participants. This information was self-reported and collected according to the signed consent by the clinic staff enrolling participants in the study. Participants were not questioned about gender, nor was this information used in the study. The results in this study apply to both sexes and also point out to various differences in multiple sclerosis disease mechanisms between females and males.<br>The effect of sex was analyzed on two levels - first, we identified physiological sex differences in protein levels in the population of healthy individuals. These physiological sex differences were regressed out from protein levels of all participants. Next, we investigated sex effects on protein levels adjusted for physiological sex differences in the cohort of people with multiple sclerosis using as a covariate in various linear models.
Reporting on race, ethnicity, or other socially relevant groupings	We have collected self-reported race data on participants as part of enrollment according to the signed consent. We have tested and reported effects of race on protein levels and their association with multiple sclerosis disease mechanisms in this study.
Population characteristics	Two populations of participants were recruited for the study: 1. population of healthy individuals, aged 19-71, with no known history of neuroimmunological diseases, and 2. population of participants with a diagnosis of multiple sclerosis (MS), aged 18-75. Longitudinal cerebrospinal fluid samples were collected, the first one generally in untreated stage. Out of 971 MS CSF samples, 65.3% were collected in untreated stage. The remaining 44.7% samples were collected from participants receiving some form of immunomodulatory therapy.
Recruitment	Participants were prospectively recruited as part of the natural history protocol. Participants were either referred to the National Institutes of Health (NIH) by their private neurologist or they were self-referred. Two potential biases could be considered in recruitment of MS participants - underrepresentation of African-american participants and over representation of females. None of these potential biases were present in our study. NIH clinical center is nation-wide biomedical research hospital funded by USA federal government. It recruits subjects interested to participate in research from entire USA.
Ethics oversight	The study protocol was approved by approved by the Institutional Review Board of the National Institutes of Health (NIH).

Note that full information on the approval of the study protocol must also be provided in the manuscript.

Field-specific reporting

Please select the one below that is the best fit for your research. If you are not sure, read the appropriate sections before making your selection.

☒ Life sciences ☐ Behavioural & social sciences ☐ Ecological, evolutionary & environmental sciences

For a reference copy of the document with all sections, see nature.com/documents/nr-reporting-summary-flat.pdf

Life sciences study design

All studies must disclose on these points even when the disclosure is negative.

Sample size	No formal samples size calculation was performed. We have analyzed a total of 1040 cerebrospinal fluid samples collected over two decades from 69 healthy individuals and 394 participants with multiple sclerosis with deeply phenotyped disease characteristics at the time of sample collection. To our knowledge, this is the largest cohort of participants/samples of its kind in the world.
Data exclusions	Two types of outlier analyses were performed: 1. In the cohort of healthy participants that was used to regress out physiological aging and sexual dimorphism effects, we eliminated outliers that fell outside the range of cohort median +/- 1.5 * interquartile range (IQR) for each of the measured protein levels (assuming that in a relatively homogeneous population of participants in the absence of neuroimmunological disease, the outliers are most likely a result of a technical bias). 2. In the cohort of multiple sclerosis (MS) samples, that were adjusted for healthy aging and sex differences, we identified outliers as values outside of the range of MS cohort median +/- 3 * IQR. These outlier values were then "floored" to the minimum or maximum of the range of MS cohort for each particular protein. The purpose of this operation was to eliminate extreme outlier values that would negatively effect downstream linear models, depending on normally distributed datasets. The

	outlier values were not eliminated completely in the MS cohort under the assumption, that unlike in healthy participants, the heterogeneity of the MS disease processes can result in outlier values, rather than all of them being attributed to technical noise
Replication	Due to the cost of the Somascan assay, all samples were analyzed only once, although Somascan assay has an excellent technical characteristics at the CV~5%. A potential technical bias was addressed as described above in the data exclusion section.
Randomization	This was a prospective study investigating natural history of multiple sclerosis, with no experimental groups and therefore no randomization. The elastic net model predicting number of contrast enhancing lesions, described in this study, was generated in a training cohort of samples, and tested in an independent cohort of samples. The randomization of the cohort into training and validation took in to account balance of disease subtypes (relapsing-remitting, primary-, and secondary progressive multiple sclerosis).
Blinding	Clinical, imaging and demographic data were collected prospectively before the samples were sent for the proteomic Somascan analysis. The data were QC-ed and locked in the research database. Somascan assay was performed on coded samples by Somalogic personnel blinded to any metadata associated with the samples.

Reporting for specific materials, systems and methods

We require information from authors about some types of materials, experimental systems and methods used in many studies. Here, indicate whether each material, system or method listed is relevant to your study. If you are not sure if a list item applies to your research, read the appropriate section before selecting a response.

Materials & experimental systems

n/a	Involved in the study	
☐	☒	Antibodies
☒	☐	Eukaryotic cell lines
☒	☐	Palaeontology and archaeology
☒	☐	Animals and other organisms
☐	☒	Clinical data
☒	☐	Dual use research of concern
☒	☐	Plants

Methods

n/a	Involved in the study	
☒	☐	ChIP-seq
☒	☐	Flow cytometry
☐	☒	MRI-based neuroimaging

Antibodies

Antibodies used	Levels of neurofilament light chain (NFL) were quantified using spectrophotometric assays by UmanDiagnostics (catalog# 10-7002)
Validation	This is a validated commercial assay.

Clinical data

Policy information about clinical studies

All manuscripts should comply with the ICMJE guidelines for publication of clinical research and a completed CONSORT checklist must be included with all submissions.

Clinical trial registration	NCT00794352
Study protocol	Study protocol can be downloaded from the NIH IRB website and made available to peer reviewers
Data collection	Data were prospectively collected between January 1999 and August 2024 at the National Institutes of Health, Bethesda, MD.
Outcomes	The primary and secondary outcomes are defined in the Natural History study protocol. The primary outcome is a definitive diagnosis of MS or other disorder. Secondary outcomes relevant to the manuscript are clinical disability measured by NeurEx and CombiWISE (see the protocol for details). The MRI outcome T2 lesion load volume was based on MRI volumetric data generated by Qmenta platform (https://www.qmenta.com) from structural images obtained at NIH. All other outcomes were generated as described in the methods section of the manuscript.

Plants

Seed stocks

Report on the source of all seed stocks or other plant material used. If applicable, state the seed stock centre and catalogue number. If plant specimens were collected from the field, describe the collection location, date and sampling procedures.

Novel plant genotypes

Describe the methods by which all novel plant genotypes were produced. This includes those generated by transgenic approaches, gene editing, chemical/radiation-based mutagenesis and hybridization. For transgenic lines, describe the transformation method, the number of independent lines analyzed and the generation upon which experiments were performed. For gene-edited lines, describe the editor used, the endogenous sequence targeted for editing, the targeting guide RNA sequence (if applicable) and how the editor was applied.

Authentication

Describe any authentication procedures for each seed stock used or novel genotype generated. Describe any experiments used to assess the effect of a mutation and, where applicable, how potential secondary effects (e.g. second site T-DNA insertions, mosiacism, off-target gene editing) were examined.

Magnetic resonance imaging

Experimental design

Design type

We performed one set of structural MRI scans (T1-weighted, T2-weighted images) at each clinic visit for each subject.

Design specifications

NA

Behavioral performance measures

NA

Acquisition

Imaging type(s)

structural

Field strength

1.5T and 3T

Sequence & imaging parameters

T1 magnetization-prepared rapid gradient-echo (MPRAGE) or fast spoiled gradient-echo (FSPGR) and T2 weighted three-dimensional fluid attenuation inversion recovery (3D FLAIR).

Area of acquisition

whole brain

Diffusion MRI ☐ Used ☒ Not used

Preprocessing

Preprocessing software

Raw unprocessed but locally anonymized and encrypted T1 - MPRAGE or T1 - FSPGR and T2 - 3D FLAIR DICOM files as input sequences, ideally with 1 mm3 isotropic resolution, were uploaded to the QMENTA platform. LesionTOADS, now implemented into the cloud-based service, is a fully automated segmentation algorithm using multichannel MRI data. The uploaded sequences are anterior commissure-posterior commissure (ACPC) aligned, rigidly registered to each other and skull stripped (the T1 image is additionally bias-field corrected). The segmentation is performed by using an atlas-based technique combining a topological and statistical atlas resulting in computed volumes for each segmented tissue in mm3.

Normalization

NA

Normalization template

NA

Noise and artifact removal

the volumetric data were manually QC-ed for artifacts

Volume censoring

Define your software and/or method and criteria for volume censoring, and state the extent of such censoring.

Statistical modeling & inference

Model type and settings

NA

Effect(s) tested

NA

Specify type of analysis: ☐ Whole brain ☐ ROI-based ☐ Both

Statistic type for inference
(See Eklund et al. 2016)

NA

Correction

NA

Models & analysis

n/a	Involved in the study
☒	☐ Functional and/or effective connectivity
☒	☐ Graph analysis
☒	☐ Multivariate modeling or predictive analysis
